# Size-Dependent Electrical Transport Properties in Conducting Diamond Nanostripes

**DOI:** 10.3390/nano11071765

**Published:** 2021-07-06

**Authors:** Andrew F. Zhou, Elluz Pacheco, Badi Zhou, Peter X. Feng

**Affiliations:** 1 Department of Physics, Indiana University of Pennsylvania, Indiana, PA 15705, USA; mnst@iup.edu; 2 Department of Physics, University of Puerto Rico, San Juan, PR 00936, USA; elluz.pacheco@upr.edu

**Keywords:** ultrananocrystalline diamond (UNCD), nanostripe, electrical transport, electrical resistivity, conductivity

## Abstract

With the advances in nanofabrication technology, horizontally aligned and well-defined nitrogen-doped ultrananocrystalline diamond nanostripes can be fabricated with widths in the order of tens of nanometers. The study of the size-dependent electron transport properties of these nanostructures is crucial to novel electronic and electrochemical applications. In this paper, 100 nm thick n-type ultrananocrystalline diamond thin films were synthesized by microwave plasma-enhanced chemical vapor deposition method with 5% N_2_ gas in the plasma during the growth process. Then the nanostripes were fabricated using standard electron beam lithography and reactive ion etching techniques. The electrical transport properties of the free-standing single nanostripes of different widths from 75 to 150 nm and lengths from 1 to 128 μm were investigated. The study showed that the electrical resistivity of the n-type ultrananocrystalline diamond nanostripes increased dramatically with the decrease in the nanostripe width. The nanostripe resistivity was nearly doubted when the width was reduced from 150 nm to 75 nm. The size-dependent variability in conductivity could originate from the imposed diffusive scattering of the nanostripe surfaces which had a further compounding effect to reinforce the grain boundary scattering.

## 1. Introduction

Diamond possesses many superior mechanical, optical and chemical properties such as high hardness, low friction and wear rate, chemical inertness and corrosion resistance, electrochemical stability, high thermal conductivity, and high electrical insulation. Furthermore, this material is also highly compatible and transparent from infrared (IR) to ultraviolet (UV). What led to a recently renewed interest in the use of diamond was the growth of CVD diamond films for the manufacture of high-power, high-frequency, and high-temperature semiconductor devices. However, diamond thin films usually contain large diamond grains that make them difficult to fabricate into well-aligned horizontal nanostripe devices.

Ultrananocrystalline diamond (UNCD) thin films contain ultra-small diamond grains with very smooth surfaces (4–7 nm RMS roughness) which do not change with the thin film thickness. The UNCD growth uses a unique Ar (99%)/CH_4_ (1%) gas mixture that induces the production of C_2_ (C=C) dimers plus CH_x_ (x = 1, 2, 3) species, producing the 3–5 nm grains on a wafer size seeded substrate [1]. Although diamond is an electrical insulator due to its wide bandgap of 5.5 eV, the introduction of N_2_ in the growth plasma has resulted in highly conductive n-type UNCD thin films. In contrast to conventional nitrogen-doped diamond films which do not give rise to electrical conduction, the nitrogen-doped UNCD (N-UNCD) thin film brings about the preferential incorporation of nitrogen in the grain boundaries. Interestingly, with the increase in N_2_ in plasma during the growth process from 0% to 20%, the electrical resistivity of UNCD film changed from high to as low as 10^−2^ Ω·cm [2]. These conducting diamond films show strong potential for a wide range of applications in biological sensors, chemical sensors, and nanoelectromechanical (NEM) systems where the well-defined planar nanostripe (NS) devices are connected with integrated electrodes [3,4,5,6,7,8].

Nitrogen-doped UNCD thin films have shown very interesting electrical transport properties since their conductivity can be engineered with the amount of N_2_ introduced in the feed gas. During the growth process, this nitrogen is energetically favored to form bonding states which end in a lone electron pair and a carbon dangling bond, promoting grain boundary conductivity which correlates with the amount of nitrogen monotonously [9]. Recently, attempts of synthesis and fabrication of UNCD nanostripes have been explored using both the “top-down” and “bottom-up” approaches. UNCD nanostripes have been routinely fabricated, and UNCD nanostripes with widths as narrow as 25 nm have been achieved [10].

Although the electrical transport properties of the N-UNCD thin film have been discussed in several publications [11,12], it remains unclear whether their nanostripe counterparts still preserve the same intrinsic electrical transport properties as the thin films. Hence, a systematic study of the electrical transport properties of nanostripes is necessary to develop a comprehensive understanding of the conducting UNCD nanostripes, which is the key to design and optimize nanosize devices and systems.

In this letter, we report on the study of the electrical transport property of UNCD nanostripes doped with 5% N_2_ in the feed gas. Free-standing 75–150 nm wide and 100 nm thick nanostripes of various lengths from 1 to 128 μm were successfully fabricated using e-beam lithography (EBL) and reactive ion etching (RIE) techniques. The nanostripes were characterized by scanning electron microscopy (SEM), selected area diffraction pattern (SADP), and Raman spectroscopy. Electrical transport properties of the single UNCD nanostripe were measured as a function of both the nanostripe length and width, to extract the size-dependent electrical resistivity and contact resistance. The obtained size-dependent nanostripe electrical resistivities were compared with those values reported for the thin films synthesized with 5% N_2_ in the feed gas.

## 2. Materials and Methods

The nitrogen-doped UNCD thin films were grown on silicon substrates using microwave plasma-enhanced chemical vapor deposition (MPCVD) process developed at Argonne National Laboratory (ANL), as described in detail previously [1]. The seeded Si wafer was placed in the MPCVD system which was pumped down to 10^−6^ Torr. The microwave frequency for the MPCVD system was either 2.45 GHz or 915 MHz. With 2000 W plasma power, the growth was then taking place in a gas mixture of argon (89%), methane (4%), and nitrogen (5%). The chamber pressure was maintained at around 100 Torr. The substrate temperature was normally set from 400 °C to 800 °C. The UNCD film with typically 100 nm thickness and 4–7 nm RMS roughness was produced in 1-h growth.

The nitrogen-doped UNCD nanostripes were fabricated by employing electron beam lithography (EBL) and reactive ion etching (RIE) techniques [10]. In general, an electron beam resist layer was first coated on the UNCD film, followed by the direct writing of the nanostripes of different widths and lengths using an e-beam writer which formed the etch mask after development. The free-standing N-UNCD nanostripes between two support pads were fabricated using an inductively coupled plasma (ICP) RIE system, including the removal of the Si substrate underneath. For the electrical property characterization, 10/100 nm of Cr/Au metal pads were aligned and patterned on the nanostripe’s supporting pads. The widths of the UNCD nanostripes were in the range of 75–150 nm with various lengths of 1–128 μm and the height of the nanostripes, determined initially by the thin film thickness, was 100 nm.

**Figure 1:**
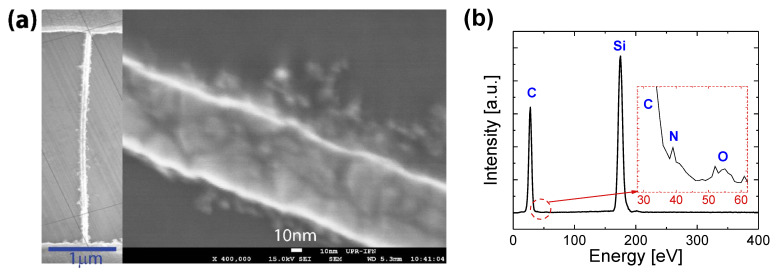
(**a**) SEM image of an N-UNCD nanostripe between two support pads. Inset: the 75 nm wide free-standing N-UNCD nanostripe. (**b**) EDS spectrum of the nitrogen-doped UNCD nanostripes.

Figure 1a shows a scanning electron microscopy (SEM) image of a single free-standing nanostripe. In addition, the energy-dispersive X-ray spectroscopy (EDS) analysis was also used to confirm the elemental composition of the nitrogen-doped UNCD nanostripe, as shown in Figure 1b. The data generated by EDX analysis consist of peaks corresponding to the elements making up the true composition of the sample being analyzed, including C, N, O, and Si. The X-ray intensity variations indicate a great concentration of carbon while the silicon peak is from the SiO_2_ sacrificial layer below the UNCD nanostripes.

**Figure 2:**
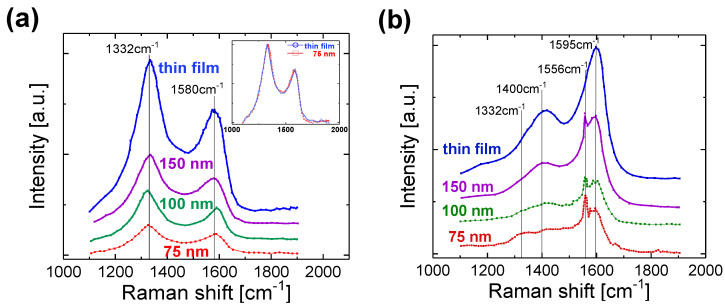
Raman spectra of the as-grown N-UNCD thin film and nanostripes of different widths recorded with laser excitation at (**a**) 633 nm, and (**b**) 325 nm.

Raman spectroscopy is the most widely used non-destructive chemical analysis technique which provides detailed information about the N-UNCD material quality. To characterize UNCD nanostripe at a global level, confocal Raman microscopy was employed at both 633 and 325 nm wavelengths. As shown in Figure 2, it is clear that as the nanostripe width scales down, the intensity of Raman spectra decreases in both visible and UV Raman spectra since the nanostripe width is much smaller than the excitation laser spot size (dia. ≥ 1 μm). After normalization, as shown by the inset in Figure 2a, for example, the Raman intensity curve of 75 nm wide nanostripe matches closely to that of the thin film. Each individual Raman spectrum shows typical shapes with two broad bands centered at 1332 cm^−1^ and 1580 cm^−1^, which demonstrate intrinsic N-UNCD signatures which are assigned to the D and G peak of sp^2^-bonded carbon, respectively [13]. The spectra profiles remain consistent, neither peak shift nor peak intensity ratio change was observed.

In the UV Raman spectra (Figure 2b), the intensity of the G peak increases with the decrease in laser excitation wavelength, resulting from the resonance Raman enhancement effect in the p-p* transition in sp^2^-bonded carbon. Moreover, the position of the D peak shifts to about 1400 cm^−1^ for all the N-UNCD samples [14]. Similar to the visible Raman spectra, an intensity decrease is also observed in the UV Raman spectra when the width decreases, besides a small peak at 1556 cm^−1^ due to Raman scattering from atmospheric and physisorbed oxygen on the sample surface [15,16]. Based on the SEM and Raman measurement, no evidence of graphitization or chemical modification in UNCD nanostripes was detected after the EBL and RIE processing, regardless of their widths.

To carry out the electrical measurements at the nanoscale, a micromanipulated four-probe station was used. The standard features of the apparatus and methods used in the present work have been described in detail previously [17], and only a brief description is given here. The UNCD nanostripe sample was mounted on the stage, and its temperature could be controlled by a resistive heater located underneath. The contact tips, made of platinum/iridium (Pt/Ir) and sharpened chemically, had a tip radius of fewer than 1 μm. The tip was precisely controlled in three dimensions and gently placed on the UNCD nanostripe metal pads with a precision better than 100 nm in each dimension to form an electrical contact. The current-voltage (I-V) measurements of the UNCD nanostripes of different widths and lengths were conducted systematically. Keithley Model 6430 sub-femtoampere source meter (Keithley Instruments, Solon, Ohio, USA) was utilized to provide the voltage from −5.0 V to 5.0 V with 0.1 V interval and to read the current signal simultaneously. No thermal annealing was performed after metal contact deposition.

## 3. Results and Discussion

**Figure 3:**
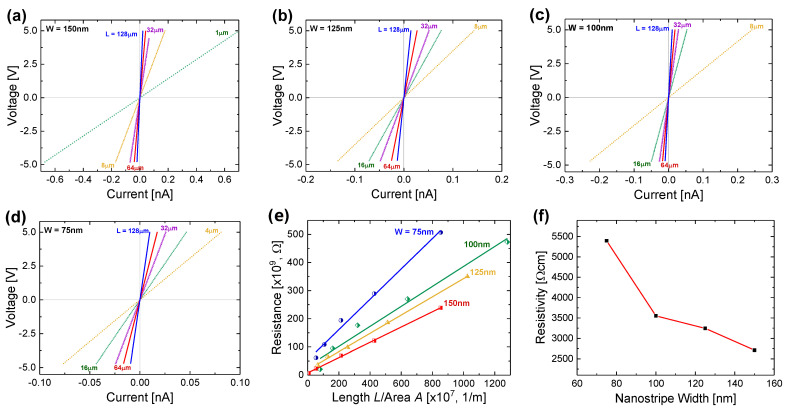
The typical V−I characteristics of (**a**) 150 nm, (**b**) 125 nm, (**c**) 100 nm and (**d**) 75 nm wide N−UNCD nanostripes of various lengths measured at room temperature. (**e**) The measured resistance as a function of the ratio of the nanostripe length/cross-section for nanostripes of different widths. The solid line is the linear fitting. (**f**) The electrical resistivity as a function of the nanostripe width after the contact resistance has been deducted.

The nanostripe’s resistivity was extracted from a transmission line method (TLM) analysis. Figure 3a–d show the typical V-I plots measured at room temperature on 150, 125, 100 and 75 nm wide N-UNCD nanostripes with lengths varying from 1 to 128 μm. The linear relationship demonstrates that the as-deposited Cr/Au produces ohmic contacts. For nanostripes of different lengths, all show a measured current directly proportional to the applied voltage. To extract the resistivity of the UNCD nanostripes and the contact resistance of the metal pad, the measured total resistance of the nanostripes is plotted as a function of the ratio of the nanostripe length *L* to the cross-sectional area *A*, i.e., *L/A*. The measured total resistance, *R_T_*, is given by the relation [18],

(1) $$R_{T}=R_{NS}+2R_{C}+2R_{M}=\rho \frac{L}{W\times H}+2R_{C}+2R_{M}$$

where *R_NS_* = *ρL*/(*W × H*) is the nanostripe resistance, *R_C_* is the contact resistance, R_M_ is the resistance of the contact metal, *ρ* is the resistivity of the UNCD, *L*, *W* and *H* are the length, width, and height of the nanostripe, respectively. For simplicity, we assume here that the cross-sectional area of a nanostripe, *A* = *W × H,* approximately, and metal electrode resistance R_M_ ~ 0 because it is far less than the other two terms. Figure 3e shows the linear plot of *R_T_* as a function of the *L*/*A* ratio. A least-squares fit to the data produced an extracted resistivity *ρ* = 2.72 × 10^3^ Ω cm for 150 nm wide nanostripe after the contact resistance has been deducted. The same process has been repeated for the N-UNCD nanostripes of different widths of 125 nm, 100 nm, and 75 nm, fabricated in the same batch, with different lengths in the range of 1–128 μm. Figure 3f shows the extracted electrical resistivity, as a function of the nanostripe width. The resistivity decreases approximately half from 5400 Ω cm to 2720 Ω cm when the nanostripe width increases from 75 to 150 nm.

The resistivity data from the N-UNCD nanostripes doped with 5% N_2_ present in the feed gas are compared with those values reported for N-UNCD thin films of the same level of N_2_ doping. Table 1 lists the reported resistivity *ρ*, or the conductivity *σ* (=1/*ρ*), of N-UNCD films doped with 5% N_2_ present in the plasma. Although the resistivity *ρ* for N-UNCD films varies from 0.009–590.2 Ω cm as reported by different groups [9,19,20,21,22,23], both 150 nm and 75 nm wide nanostripes have much higher resistivities than these reported values for the thin film counterparts. As shown in Table 1, the resistivity of 75 nm wide nanostripe is about 100 times larger than the reported resistivity of an N-UNCD thin film doped with 5% N_2_ in the reactor.

It is evident that the electrical transport property of N-UNCD nanostripes depends on the nanostripe dimensions. It has been reported that the resistivity of metallic thin films and nanostripes increased drastically when the film thickness or wire diameter diminished [18]. To explain the size-dependent electrical resistivity, we adopted the electron scattering model estimated by Matthiessen’s rule [24],

(2) $$\rho_{Total}=\rho_{0}+∆\rho_{FS}+∆\rho_{MS}$$

where *ρ*_0_ is the bulk resistivity, **∆***ρ_FS_* is the scattering contribution by surface (Fuchs-Sondheimer, FS) mode and **∆***ρ_MS_* is the scattering contribution by grain boundary (Mayadas-Shatzkes, MS) mode. In N-UNCD, since the electron transport occurs between insulating grains, the nanostripe with a smaller width will introduce more diffusive scattering with the nanostripe surfaces, leading to an increase in FS mode resistivity. In addition, the diffusive scattering with the nanostripe surfaces will further enhance the grain boundary scattering which also increases the MS mode resistivity. Therefore, the electrical resistivity increases when the nanostripe width decreases, as observed.

## 4. Conclusions

In summary, the electrical transport properties of a single N-UNCD nanostripe were measured as a function of both nanostripe width and the ratio of length to cross-section. The resistivity of 75 nm wide N-UNCD with 5% N_2_ was measured by the transmission line method, which was on the order of 5.40 × 10^3^, at least one order of magnitude larger than that of a thin film counterpart. The size-dependent of electrical transport properties were observed in diamond nanostripes for the first time and the results are crucial to design and build N-UNCD nanostripe based nanoelectronic devices, miniature chemical and biological sensors.

## Figures and Tables

**Table 1:** Comparison of the room temperature electrical conductivity/resistivity of the thin film and nanostripe of UNCD doped with 5% nitrogen presented in the plasma.

5% N_2_ Present in Plasma	Conductivity *σ* (Ω cm)^−1^	Resistivity *ρ* (Ω cm)	Reference
Thin Film	0.1	(10)	[9]
Thin Film	13.20	(0.0758)	[19]
Thin Film	(1.69 × 10^−3^–111)	0.009–590.2	[20]
Thin Film	0.14	(7.14)	[21]
Thin Film	7.5	(0.133)	[22]
Thin Film	0.01	(100)	[23]
150 nm wide NS	3.68 × 10^−4^	2.72 × 10^3^	This work
75 nm wide NS	1.85 × 10^−4^	5.40 × 10^3^	This work

## Data Availability

The data presented in this study are available on request from the corresponding authors.
